# Onsite production of medical air: is purity a problem?

**DOI:** 10.1186/s40248-018-0125-8

**Published:** 2018-05-07

**Authors:** Paul Edwards, Patricia-Ann Therriault, Ira Katz

**Affiliations:** 1 VitalAire Canada Inc., Mississauga, ON L5N 8R9 Canada; 2 Medcial R&D, Air Liquide Santé International, 78354 Les Loges-en-Josas, France; 3 Department of Mechanical Engineering, Lafayette College, Easton, PA 18042 USA

**Keywords:** Medical air, Risk assessment, Carbon dioxide

## Abstract

**Introduction:**

Medical air (MA) is widely used in hospitals, often manufactured onsite by compressing external ambient air and supplied through a local network piping system. Onsite production gives rise to a risk of impurities that are governed by the same pharmacopoeia purity standards applicable to commercially produced MA. The question to be addressed in this paper is how to assess if a lack of purity poses a medical problem?

**Methods:**

The MA produced onsite at a major Canadian hospital was monitored for carbon dioxide (CO_2_) and other impurity gases at high frequency (one per minute) over a two-month period.

**Results:**

The average CO_2_ concentration was 255 ppm. The United States Pharmacopeia (USP) threshold of 500 ppm was exceeded during 1% of the total study period, and the average while exceeding the threshold was 526 ppm. The maximum concentration was 634 ppm.

**Discussion and conclusion:**

To our knowledge, there is only one study that evaluated the effects suffered by respiratory patients of elevated nitric oxide in MA; thus, it is not clear what are the medical bases for the thresholds stated in the USP. To perform a Quality Risk Assessment, the threshold and the time above threshold should be considered in determining the frequency of sampling and analysis, and operating methods required to ensure the quality of MA entering the pipeline meets the clinical, regulatory, and patient safety standards. In conclusion, because the USP does not provide impurity thresholds for specific patients nor time above thresholds, there is a need for the medical community to determine these quantities before it can be known if the purity of MA is a problem.

## Introduction

Medical air (MA) is widely used for aerosol drug delivery, high flow therapy, mechanical ventilation, neonatal environment control, infant resuscitation, general anesthesia, and hyperbaric therapy. MA can be produced by external manufacturers, filled in cylinders and transported to the site of use. This form of supply is directly regulated by the relevant country’s pharmacopeia [1]. However, for hospitals MA is most efficiently manufactured onsite by compressing external ambient air and supplied through a local network piping system. Onsite production gives rise to a risk of impurities that are governed by the same pharmacopoeia purity standards, and should be assessed by the facility’s staff [2]. The sources of impurities can be categorized as systemic or extrinsic; where the systemic sources arise due to the manufacturing and distribution systems themselves and extrinsic sources arise from the external air input. Furthermore, the extrinsic sources are either endemic (i.e., local to the production site) or ambient (arising from the pollution levels in the environment).

In brief, onsite MA production consists of outside air being drawn into the system by a compressor, through particulate filters. The MA passes through a dryer before entering the distribution network. There also must be a backup supply based on cylinders. Note that there would usually be multiple components such as compressors and dryers to optimize production and to account for normal downtime. Of particular importance are dryers that consist of dual columns that operate on the principle of pressure swing adsorption, when one column swings to low pressure to dehumidify the absorbing material and the second takes over as the active dryer.

For context, the United States Pharmacopeia (USP) threshold values for MA are: oxygen < 19.5% v/v and > 23.5% v/v, carbon monoxide > 10 ppm, carbon dioxide > 500 ppm, nitrous oxide + nitric oxide > 2.5 ppm, sulphur dioxide > 5 ppm, and humidity <− 5 °C pressure dew point [3].

The question to be addressed in this paper is how to assess the purity of onsite produced MA and if a lack of purity poses a medical problem. That is, what are the elements of a Quality Risk Assessment for onsite produced MA that should be understood and addressed by the medical community. We take advantage of a unique sample dataset; high frequency measurements (one per minute) over a two-month period of carbon dioxide (CO_2_) to frame the discussion.

## Methods

The MA produced onsite at a major Canadian hospital (the name of the hospital cannot be revealed for contractual reasons) was monitored using an AerAlin device (Vitalaire, Mississauga, Canada) that monitors the oxygen content and contaminant levels. Using seven independent sensors the AerAlin can monitor the following USP designated components: oxygen, carbon dioxide, carbon monoxide (CO), dewpoint (to determine humidity), nitric oxide and nitrogen dioxide together (NOx), and sulphur dioxide (SO_2_). In this short paper we only report in detail on CO_2_ results. The internal CO_2_ sensor (Vaisala CARBOCAP series GMT220, Helsinki, Finland) has an accuracy: ± (1.5% of range + 2% of reading). This sensor has been used in a wide variety of scientific and industrial activities [4–6]. Measurements were recorded at a frequency of 1/min, from July 28th to September 12th, 2017.

## Results

**Fig. 1:**
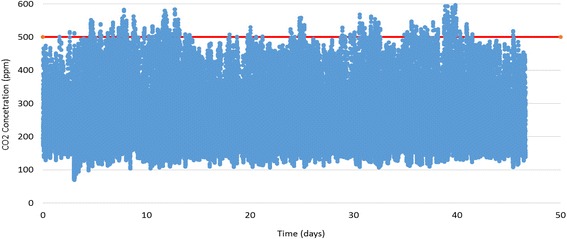
The time history of CO_2_ concentration. The USP threshold of 500 ppm is shown in red

Figure 1 shows the time history of CO_2_ concentration during the study period. The red horizontal line is the USP threshold. The average CO_2_ concentration was 255 ppm. The USP threshold of 500 ppm was exceeded during 1% of the total study period (719 measurements), and the average while exceeding the threshold was 526 ppm. The maximum concentration was 634 ppm.

Considering the other USP criteria; there were nine threshold breaches of NOx (maximum 2.7 ppm) with an overall average of 0.03 ppm. There were no threshold breaches for the other USP criteria: O_2_ (avg 21.0%, max 21.1%, min 20.9%), CO (avg 1.7 ppm, max 9.6 ppm), dew point (avg − 42.1 °C, max 8.3 °C), and SO_2_ (avg < 0.001 ppm, max 0.1 ppm).

## Discussion and conclusion

In this paper a dataset of CO_2_ concentration measurements in onsite produced MA indicates periodic breaches in the USP threshold. To our knowledge, this is the first example of testing of onsite production of MA to appear in the peer reviewed literature.

The reason for the breaches in CO_2_ threshold are attributed by us to the dryer, which captures CO_2_ concomitant to humidity. When it is almost saturated with humidity, before the swing to purge mode, previously captured CO_2_ is released into the airstream at higher concentrations than in the original supply. Thus, this is considered a systematic impurity source.

We believe this example of CO_2_ concentration presented herein showing periodic systematic impurity breaches is representative of potential extrinsic impurity problems, though during the measurement period at this site there were only the nine other threshold breaches of NOx (maximum 2.7 ppm).

While there are numerous studies confirming the health effects of common air pollutants [7], to our knowledge there is only one study that evaluated the effects suffered by respiratory patients inhaling contaminated MA. This study showed that unintended inhalation of NO in industrialized areas may alter the PaO_2_ and may make the therapeutic use of NO less successful [8]. Another study concluded that low levels of carboxyhemoglobin (due to breathing CO) exacerbate myocardial ischemia during graded exercise in subjects with coronary artery disease [9]. Thus, overall it is not clear what are the medical bases for the thresholds stated in the USP.

To perform a Quality Risk Assessment, the threshold and the time above threshold should be considered in determining the frequency of sampling and analysis, and operating methods required to ensure the quality of MA entering the pipeline meets the clinical, regulatory, and patient safety standards. In the greater context of MA provided in cylinders, recalls have been made for levels of 720 PPM [10].

In conclusion, because the USP does not provide impurity thresholds for specific patients nor time above thresholds, there is a need for the medical community to determine these quantities before it can be known if the purity of MA is a problem.
